# Res﻿earch on dust dispersion law of fully mechanized mining faces under different inclinations and tracking closed dust control method

**DOI:** 10.1038/s41598-022-20606-9

**Published:** 2022-10-05

**Authors:** Gang Zhou, Yang Kong, Qunzhi Meng, Bingyou Jiang, Yongwei Liu, Gang Li, Biao Sun, Jinli Wang, Dong Yan, Zhenhua Li

**Affiliations:** 1 College of Safety and Environmental Engineering, Shandong University of Science and Technology, Qingdao, 266590 China; 2 State Key Laboratory of Mining Disaster Prevention and Control Co-founded by Shandong Province and the Ministry of Science and Technology, Shandong University of Science and Technology, Qingdao, 266590 China; 3 Key Laboratory of Industrial Dust Prevention and Control & Occupational Safety and Health, Ministry of Education, Anhui University of Science & Technology, Huainan, 232001 China; 4 Sinosteel Maanshan General Institute of Mining Research Co., Ltd., Maanshan, 243000 China; 5 Shanghai Research Institute, China Coal Technology and Engineering Group, Shanghai, 200030 China

**Keywords:** Environmental sciences, Coal

## Abstract

Based on the gas–solid two-phase flow theory, numerical simulation of the dust dispersion law of fully mechanized mining work under different inclination angles and comparative analysis of field-measured data show that with the increase of working face inclination, the inclination of airflow into the unmined zone increases from 25° to 50° and the maximum wind speed increases from 2.16 to 2.25 m/s after the mixing of cutting turbulent wind and system ventilation. Meanwhile, the range of high-concentration dust clusters, suspension time, lateral migration intensity, and deposition zone increase to varying degrees; dust clusters increases from 62.02 to 202.46 m^3^. When X < 53.96 m, the dust concentration in the sidewalk-breathing zone shows a sine function with the length of the working face, and when X ≥ 53.96 m, it satisfies the exponential decay function. Based on this, the tracking closed dust control technology is proposed. Combining the offset angle of the airflow and t the gathering position of dust mass, the wind curtain angle and air velocity are automatically controlled to ensure that the dust is restricted to one side of the cable trough.

## Introduction

**Figure 1:**
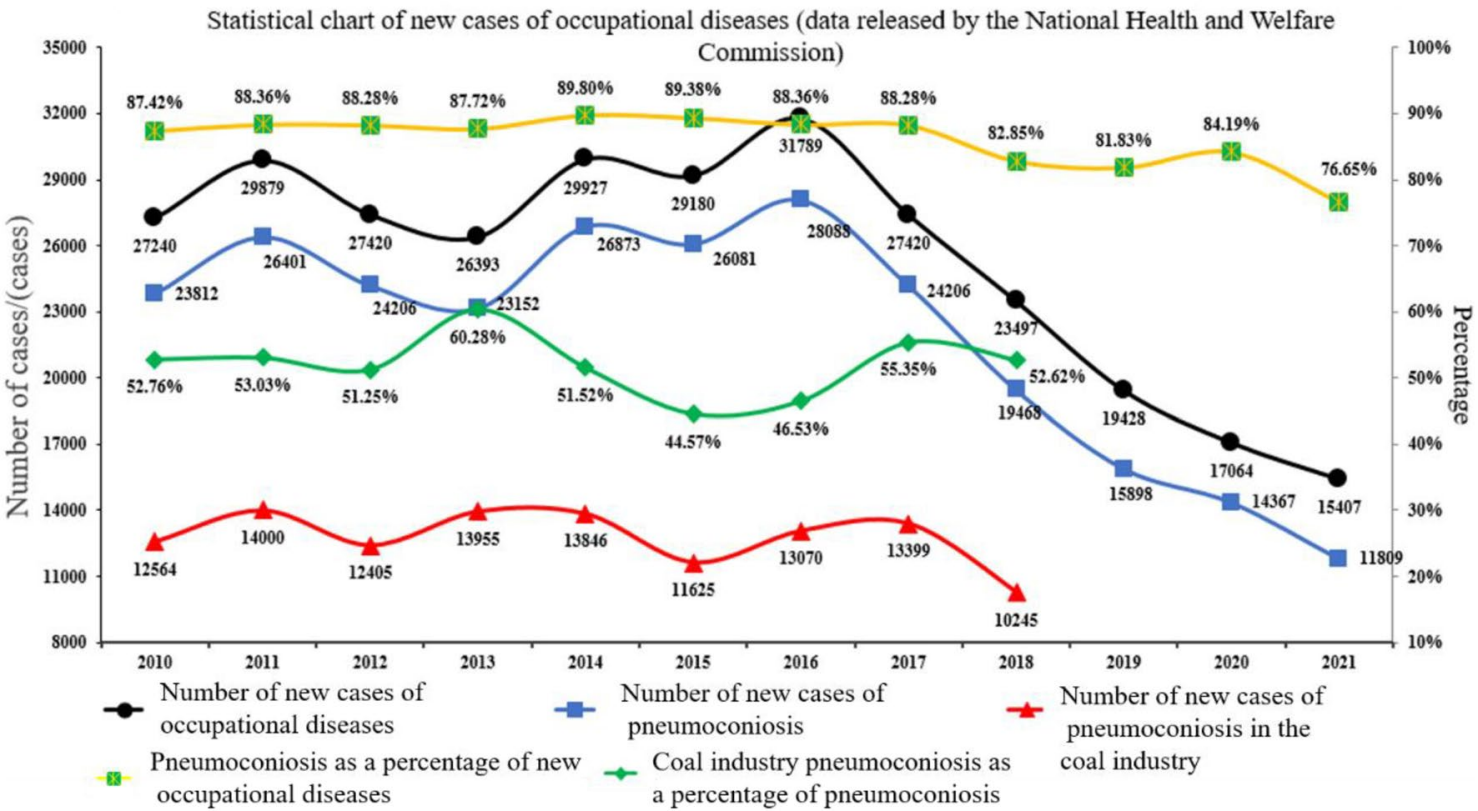
Statistics of occupational disease cases.

With increasing levels of mechanization, coal production has increased annually, and respirable dust has accumulated on coal mining faces. High concentrations of dust can cause pneumoconiosis, reduce the accuracy of instrument work, and cause coal and gas explosions, threatening the physical and mental health of workers^1–4^. According to incomplete statistics, by the end of 2021 China had 11,809 cases of pneumoconiosis, and between 2010 and 2021, accounting for the total occupational pneumoconiosis are as high as 80% a year, more than 50% by coal dust induced^5,6^ (Fig. 1). In addition, due to the increase in coal dust concentration, the dust explosion pressure and explosion index first increased and then decreased. According to existing statistics, 87.32% of China’s 532 key coal mines are at risk of coal dust explosions^7–9^. The highest concentration of coal dust pollution is in underground working environments. The dust production is approximately 45%–80% of the total dust production of the mine^10–12^. However, although most mines have adopted various air dust removal measures, the dust concentration on the working face far exceeds the relevant regulations in the “Coal Mine Safety Regulations”. In long-term exploration, controlling flour dust in comprehensive mining work is difficult, and the dust concentration is high. Many on-site dust removal systems and equipment arrangements cannot achieve the intended effect or cannot be used at key points. Therefore, studying the dispersion law of flour dust in coal mining is of substantial practical significance in solving the excessive dust concentration, implementing dust-proof technical solutions, and ensuring safe production in coal mines.

Numerical simulation is fast and effective, has the obvious visualization, and has the advantage of a detailed analysis in each zone. Many scholars have used numerical simulations to study the law of dust dissipation. Pathankar et al. used the Lagrangian method to describe the movement of dust particles and analyzed the migration behavior of dust particles under different Stokes numbers driven by airflow^13^. Hossein and Gholamreza used computational fluid dynamics to determine the settlement position of dust accumulation zones and particle sizes of different sizes on the working face, improving the mine ventilation system and the sanitary conditions of the working face^14^. Zhang et al., based on computational fluid dynamics and the finite volume method, analyzed the diffusion and pollution characteristics of respirable dust in different zones and different dust sources on the long-wall working face at the macro and micro scales^15^. Based on the gas–solid two-phase coupling model, Yao et al. studied the combined movement of coal dust vertical displacement, strike horizontal displacement, and inclined horizontal displacement in a large-dip, steeply inclined, fully mechanized caving face; the results showed found that when the fully mechanized caving face had a large inclination angle, the wind flow was turbulent, the air velocity was high, and the movement time on the working surface was long^16,17^. Based on computational fluid dynamics, Hu, Liao et al., and Cai, Nie et al. have studied dust migration laws under different air velocities and air volumes and found that when the dust concentration is relatively low, the increase in airflow leads to dust entrainment^18,19^. Zhang et al. found that the airflow velocity distribution, the dust migration trajectory, and the influence of airflow on dust diffusion when cutting coal with downwind differs from those when cutting coal with upwind. Thus, the dust reduction method was optimized, and dust reduction efficiency was improved^20^. Lu Yuezea and Akhtar Saad used computational fluid dynamics to evaluate various possible situations of underground mine configurations and found that the presence of continuous coal mining machines adversely affected airflow and increased methane and dust concentrations. This negative impact can be achieved by minimizing or neutralizing the operation of the scrubber fan in the suction mode^21^. Lu et al. used the Reynolds stress model and the discrete phase model to study the deposition characteristics of soot particles in an inclined heat exchange channel with surface fins. The results showed that the particle diameter and flue gas flow rate affected the ribbed channel. The deposition efficiency has a significant impact. The dip angle has little effect on the deposition efficiency of small particles, but has a significant impact on the deposition efficiency of large particles^22^.

However, most scholars have not considered the influence of face angle on airflow and coal dust dispersion characteristics, resulting in errors in their research results, and thus could not provide a theoretical basis for the prevention and control of dust in multiple coal mines on a large scale to improve comprehensive mining. To fill this gap in the literature, this study conducted basic research on airflow and coal dust pollution characteristics under different inclination angles of the working face. In this study, the 3604 working face of Feicheng Mining Group Shanxian Energy Co., Ltd. was used as the prototype; the working face inclination was the only variable; and one of the nearly horizontal, gently inclined, inclined, and steeply inclined working faces was used to establish a gas–solid two-phase flow model. FLUENT (version 2020R1) numerical simulation software was used to conduct detailed research on the downwind movement and coal dust pollution characteristics at different inclination angles of a fully mechanized mining face an analysis of the data obtained and an air curtain tracking closed dust control technology scheme is proposed to provide a theoretical basis for the comprehensive management of coal mining working face dust.

## Mathematical model

The mathematical model in this study consists of the Navier–Stokes equation (NS; the Eulerian method) in polar coordinates^23^. For turbulent flow, the standard k–ε two-equation model was used. The Lagrangian method and discrete phase model were used to solve the dispersion law of working flour dust.

### Wind flow model

The k–ε equation model based on the Reynolds time-averaged NS has been widely used in studying the dispersion of complex particles. Suppose u, v, and w are the velocity components in the x-, y-, and z-directions, respectively. Therefore, the speed is expressed as the sum of the instantaneous pulsating speed and time-averaged speed^24–26^

1 $${\text{u}} = \overline{u} + u^{\prime } \, {\text{v}} = {\overline{\text{v}}} + {\text{v}}^{\prime } \,{\text{w}} = {\overline{\text{w}}} + {\text{w}}^{\prime }$$

Generally, the airflow state of the working surface is regarded as an incompressible fluid, and the continuity equation is^27,28^

2 $$\frac{{\partial\uprho }}{{\partial {\text{t}}}} + \frac{{\partial\uprho {\text{u}}_{{\text{i}}} }}{{\partial {\text{x}}_{{\text{i}}} }} = 0$$

where ρ is the gas density (kg/m^3^), and t is the time. When the airflow is in a steady state, the density does not change with time and can be written as

3 $$\frac{{\partial \left( {\uprho {\text{u}}} \right)}}{{\partial {\text{x}}}} + \frac{{\partial \left( {\uprho {\text{v}}} \right)}}{{\partial {\text{y}}}} + \frac{{\partial \left( {\uprho {\text{w}}} \right)}}{{\partial {\text{z}}}} = 0$$

The Reynolds time-averaged NS equation is used to derive and calculate the renormalized k–ε equation using the mathematical method of renormalization.

The $k$ equation is^29,30^

4 $$\frac{{\partial \left( {\uprho {\text{k}}} \right)}}{{\partial {\text{t}}}} + \frac{{\partial \left( {\uprho {\text{ku}}_{{\text{i}}} } \right)}}{{\partial {\text{x}}_{{\text{i}}} }} = \frac{\partial }{{\partial {\text{x}}_{{\text{j}}} }}\left[ {\left( {\upalpha _{{\text{k}}}\upmu _{{{\text{eff}}}} } \right)\frac{{\partial {\text{k}}}}{{\partial {\text{x}}_{{\text{j}}} }}} \right] + {\text{G}}_{{\text{k}}} + {\text{G}}_{{\text{b}}} - {\uprho \varepsilon }$$

In the formula, *k* is the turbulent kinetic energy, m^2^/s^2^; $\alpha_{k}$ is the reciprocal of the effective Prandtl number of the turbulent kinetic energy, that is, $\alpha_{k} = \frac{1}{{\sigma_{k} }} = 1.0$; $\mu_{eff}$ is the viscosity coefficient; $G_{k}$ is the turbulent kinetic energy caused by the average velocity gradient; and $G_{b}$ is the turbulent kinetic energy caused by the influence of buoyancy.

The ε equation is^31,32^

5 $$\frac{{\partial\uprho {\text{k}}}}{{\partial {\text{t}}}} + \frac{{\partial \left( {{\uprho \varepsilon }{\text{u}}_{{\text{i}}} } \right)}}{{\partial {\text{x}}_{{\text{i}}} }} = \frac{\partial }{{\partial {\text{x}}_{{\text{j}}} }}\left[ {\left( {\upalpha _{\upvarepsilon } {\upmu }_{{{\text{eff}}}} \frac{{\partial\upvarepsilon }}{{\partial {\text{x}}_{{\text{j}}} }}} \right)} \right] + {\text{C}}_{{1\upvarepsilon }} \frac{\upvarepsilon }{{\text{k}}}\left( {{\text{G}}_{{\text{k}}} + {\text{C}}_{{3\upvarepsilon }} {\text{G}}_{{\text{b}}} } \right) - {\text{C}}_{{2\upvarepsilon }}\uprho \frac{{\upvarepsilon ^{2} }}{{\text{k}}}$$

6 $$\left\{ {\begin{array}{*{20}c} {\upmu _{{{\text{eff}}}} =\upmu _{{\text{t}}} +\upmu } \\ {\upmu _{{\text{t}}} =\uprho {\text{C}}_{\upmu } \frac{{{\text{k}}^{2} }}{\upvarepsilon }} \\ \end{array} } \right.$$

In the formula, ε is the turbulent energy dissipation rate, m^2^/s^3^; $C_{1\varepsilon }$, $C_{2\varepsilon }$, and $C_{3\varepsilon }$ are empirical constants; generally, the default is $C_{1\varepsilon } = 1.43$, $C_{2\varepsilon } = 1.91$, and $C_{3\varepsilon } = 0.09$; $\mu_{t} {\text{and}} \mu$ are the viscosity coefficients of turbulent and laminar flow; and $\alpha_{\varepsilon }$ is the reciprocal of the effective Prandtl number of the dissipation rate $\alpha_{\varepsilon } = \frac{1}{{\sigma_{\varepsilon } }} = 0.768$.

### Dust discrete model

The Euler–Lagrange method was used to calculate the idea, the main phase is described by the Euler method, the particle term is described by the Lagrangian method, and the gas–solid two-phase flow discrete phase model simulation of dust particles was used. In essence, the calculation of the trajectory of the working dust involves the integration of the differential equation of the force acting on the dust^33–35^. Therefore, the differential equations of these forces in the Cartesian coordinate system can be expressed as follows (here, the x-axis direction is considered an example)

7 $$\frac{{{\text{du}}_{{\text{p}}} }}{{{\text{dt}}}} = {\text{F}}_{{\text{D}}} \left( {{\text{u}} - {\text{u}}_{{\text{p}}} } \right) + \frac{{{\text{g}}_{{\text{x}}} \left( {\uprho _{{\text{p}}} -\uprho } \right)}}{{\uprho _{{\text{p}}} }} + \sum {\vec{\text{F}}}_{{\text{x}}}$$

In the formula, $u_{p }$ is the particle velocity, m/s; t is the time, s; $u$ is the relative velocity of the fluid, m/s; $g_{x }$ is the gravitational acceleration in the x direction, m/s^2^; $F_{D}$ is the resistance of the particle, N; $\mu$ is the hydrodynamic viscosity, Pa*s; $\rho$ is the fluid density, kg/m^3^; $\rho_{p}$ is the particle density, kg/m^3^; and $\sum {\vec{\text{F}}}_{x}$ is other forces in the $x$ direction (e.g., “apparent mass force,” thermal, the resultant force of swimming force, and Brown force).

8 $${\text{F}}_{{\text{D}}} = \frac{{18\upmu }}{{\uprho _{{\text{p}}} {\text{d}}_{{\text{p}}}^{2} }}\frac{{{\text{C}}_{{\text{D}}} {\text{Re}}}}{24}$$

9 $${\text{Re}} = \frac{{\uprho {\text{d}}_{{\text{p}}} \left| {{\text{u}}_{{\text{p}}} - {\text{u}}} \right|}}{\upmu }$$

10 $${\text{C}}_{{\text{D}}} = {\text{a}}_{1} + \frac{{{\text{a}}_{2} }}{{{\text{Re}}}} + \frac{{{\text{a}}_{3} }}{{{\text{Re}}^{2} }}$$

where ${\text{d}}_{{\text{p}}}$ is the particle diameter (m); ${\text{Re }}$ is the relative Reynolds number of the particle; ${\text{C}}_{{\text{D}}}$ is the drag coefficient; and ${\text{a}}_{1}$, ${\text{a}}_{2}$, and ${\text{a}}_{3}$ are constants within a certain Reynolds number range.

To increase the accuracy of descriptions of the movement of respirable dust particles, this study introduced a discrete element collision model to increase their suitability for field practice^36^. Using Newton’s second law, the ordinary differential equation that controls the motion of particles is expressed as follows:

11 $${\text{m}}_{{\text{p}}} \frac{{{\text{d}}\overrightarrow {{\text{v}}} }}{{{\text{dt}}}} = \vec{F}_{Drag} + \vec{F}_{Pressure} + \vec{F}_{gravitation} + \vec{F}_{Virtual - mass} + \vec{F}_{Other}$$

12 $$\overrightarrow {{\text{v}}} = \frac{{{\text{dx}}}}{{{\text{dt}}}}$$

For a given collision pair, the magnitude of the spring constant of the normal contact force should at least fulfill the following conditions: for the largest inclusion and the highest relative velocity in the collision pair, the spring constant should be sufficiently high to make the recoil of the two packages collide with the package diameter, and the maximum overlap should not be too large. The spring constant can be written as

13 $${\text{K}} = \frac{{\uppi v_{c}^{2} }}{{3\varepsilon_{D}^{2} }}D\rho$$

where ${\text{v}}_{{\text{c}}}$ is the relative velocity between two colliding particles, $\varepsilon_{D}$ is the diameter allowed to overlap, D is the package diameter, and $\rho$ is the particle mass density.

## Model establishment and related verification

### Model building

**Figure 2:**
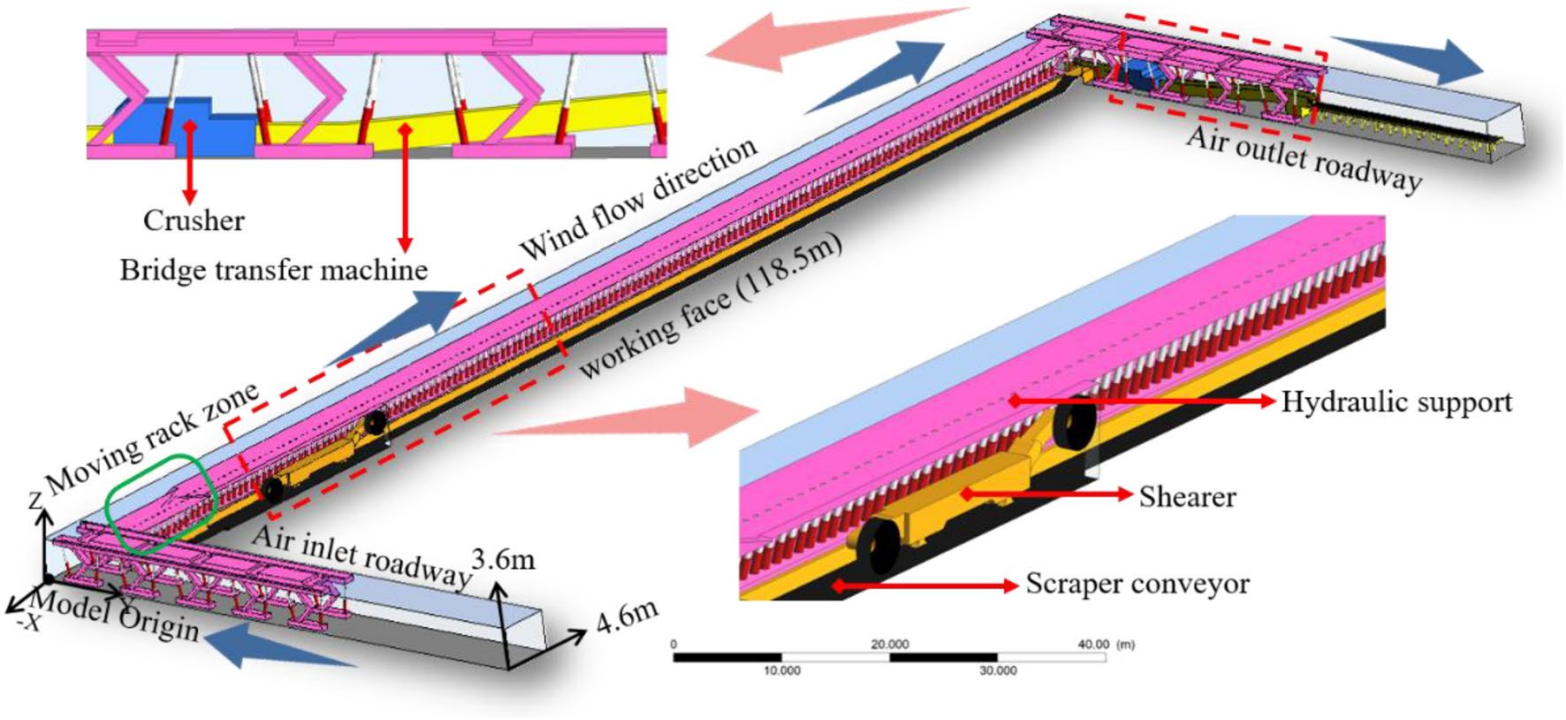
Three-dimensional model of the fully mechanized mining face.

Take the 3604 working face of Feicheng Mining Group Shanxian Energy Co., Ltd. which has a total length of 118.5 m, a net width of 6.5 m, and a maximum mining height of 3.6 m. as an example, it adopts a full-height mining process and full negative pressure. The U-shaped independent ventilation working face mining equipment comprises ZY8000/20/43 type two-pillar shielded hydraulic support, 79 frames, and the center distance of the support is 1.5 m; an MG500/1130-WD type double drum electric traction shearer with a drum diameter of 2000 mm and cutting depth of 600 mm; an SGZ800/800 type double-chain scraper conveyor; a PLM2000 wheel crusher; and a DY1000 belt conveyor. SolidWorks software was used to build a full-scale 3D mathematical model of proportional scale (Fig. 2): working surface, 118.5 m × 6.5 m × 3.6 m (length × width × height); inlet and return air lanes, 49.5 m × 4.6 m × 3.6 m (length × width × height); hydraulic support base height, 0.6 m; support center distance is 1.5 m; a total of 79 frames; shearer main body, 8.1 m × 1.2 m × 1.4 m (length × width × height); mining height, 3.6 m; drum diameter, 2000 mm; cutting depth, 600 mm; and the moving frame lags behind the 6 hydraulic supports for the rear drum of the shearer.

### Meshing and independence test

The geometric model was meshed by combining proximity and curvature size functions. First, the curvature size function was used to mesh the model as a whole; next, the approximate size function was used to further divide the mesh in order to increase the density of the neighboring size functions. Finally, 3,056,258 mesh were obtained. However, the mesh convergence should be tested for independence before numerical simulation calculation. This study was based on the grid convergence index (GCI) to test the mesh quality for independence. The main process is as follows:

1. Define a representative mesh size $l$ for the calculation:
  14 $${\text{l}} = \left[ {\frac{1}{{\text{N}}}\mathop \sum \limits_{{{\text{i}} = 1}}^{{\text{N}}} \Delta {\text{v}}_{{\text{i}}} } \right]^{1/3}$$
  where $\Delta {\text{v}}_{{\text{i}}}$ is the volume of the i unit, and N is the total number of units used in the calculation.
2. Explore the value of key variables in the simulation process under different mesh schemes (this article uses dust concentration c as the key variable).
3. Calculate the relative error between the key variables between the coarse solution and the fine solution:
  15 $$\upvarepsilon = \frac{{{\text{c}}_{{{\text{i}},{\text{coarse}}}} - {\text{c}}_{{{\text{i}},{\text{fine}}}} }}{{{\text{c}}_{{{\text{i}},{\text{fine}}}} }}$$
  where ε is the relative error, ${\text{c}}_{{{\text{i}},{\text{coarse}}}}$ is the coarse mesh convergence solution, and ${\text{c}}_{{{\text{i}},{\text{fine}}}}$ are the fine mesh convergence solutions.
4. Calculate the root mean square of the relative error metric of the critical region and multiple points ‘n(n = 1500)’ in the critical region:
  16 $$\upvarepsilon _{{{\text{rms}}}} = \left( {\frac{{\mathop \sum \nolimits_{{{\text{i}} = 1}}^{{\text{n}}}\upvarepsilon _{{\text{i}}}^{2} }}{{\text{n}}}} \right)^{1/2}$$
5. Because the mesh reduction factor is less than 2, the relative error needs to be adjusted accordingly. Additionally, to achieve the condition that the value of $\varepsilon_{rms}$ is extrapolated to the condition that the real mesh is reduced to half, the GCI should be used for the fineness grid as follows:
  17 $${\text{GCI}} = {\text{F}}\frac{{\upvarepsilon _{{{\text{rms}}}} }}{{{\text{r}}^{{\text{p}}} - 1}}$$

In Eq. (17), F is the safety factor (this study uses four sets of mesh schemes to calculate the GCI; thus, the safety factor is 1.25), ${\text{r}}$ is the mesh refinement factor, and $p$ is the convergence accuracy, which is 1.97.

Mesh was used to mesh the constructed 3D model and divide the mesh schemes (A–D, 800,000–3,000,000 units). Simultaneously, when the mesh is refined, a constant reduction value is maintained in the three coordinate directions. The meshing scheme is shown in Table 1 (a). According to the calculation program, the GCI value was calculated using the dust concentration value of 1500 points in the coal mining zone, as shown in Table 1 (b). The results show that by continuously refining the mesh, the $\varepsilon_{rms}$ and GCI values based on the dust concentration $c$ gradually decrease. A general belief is that denser the number of mesh, closer the numerical solution is to the exact solution, and smaller the truncation error and the GCI; when the GCIs of two continuous mesh are both less than 0.5%, the mesh is considered to be convergent. The GCI values under conditions C and D were 0.49% and 0.37%, respectively, and lower than the rated standards. The GCI value of scheme D is further reduced based on scheme C, indicating that the divided mesh achieves mesh independence.

**Table 1:** Detailed information on the grid schemes and grid convergence measures.

	Mesh scheme	Mesh		Min. Mesh spacing
		Cells (× 10^4^)	Vertexes (× 10^4^)	
(a)	Coarse (A)	80	20.41	0.21
	Medium (B)	160	39.76	0.17
	Fine (C)	240	58.57	0.14
	Very fine (D)	300	73.62	0.12
(b)	Mesh size (× 10^4^)	r value	${\varepsilon }_{rms}$ (%)	$\mathrm{GCI}$ (%)
	80–160	1.35	1.42	2.20
	160–240	1.35	0.32	0.49
	240–300	1.35	0.24	0.37

### Determination of boundary conditions and dust source parameters

The divided mesh was imported into FLUENT, and the parameters were set. The boundary conditions and dust source parameters set in the numerical simulation were set according to the actual operating conditions on site. The airflow velocity at the air inlet was obtained by calculating the average value after multiple measurements at the site with an anemometer. Dust source parameters were collected on site with a dust sampler and dust-containing filter membrane. The filter membrane weighing method was used to measure dust concentration, and the particle method was used to measure dust particle size. In the simulation, the downhole flowing air was regarded as an incompressible fluid, and the temperature field remained unchanged. The inlet air alley was set as the speed inlet. The return air alley was set as the pressure outlet to ensure that no backflow phenomenon occurred during the simulation. Describe the four inclinations of the working surface by changing the magnitude of the gravitational acceleration See Table 2 for detailed parameter settings.

**Table 2:** Basic parameter settings.

Project		Parameter settings
Turbulence model	k-$\upvarepsilon$	Standard
Boundary conditions	Entry boundary type	VELOCITY-INLET
	Type of export boundary	PRESSURE-OUTLET
	Inlet air velocity	1.08 m/s
	Roller axial plane	VELOCITY-INLET(1)
	Roller radial plane	VELOCITY-INLET(2)
	VELOCITY-INLET(1)	0.5 m/s
	VELOCITY-INLET(2)	-0.379 m/s
	Hydraulic diameter	4.03 m
	Turbulence intensity	3.29%
Dust source parameters	Dust generation method	surface
	Mass flow of dust produced by drum cutting	11 g/s
	Dust mass flow rate of moving rack	12 g/s
	Minimum particle size of dust	2.27 e–07 m
	Medium size of dust	4.19 e–06 m
	Maximum particle size of dust	2.93 e–05 m
	Distribution index	1.79
	Distribution method	Rosin–Rammler
Working face inclination	Near horizontal 0°	G = 9.81 m/s^2^
	Gently tilt 20°	G = 9.21 m/s^2^
	Tilt 40°	G = 7.51 m/s^2^
	60° steep	G = 4.90 m/s^2^

### Model validation

**Figure 3:**
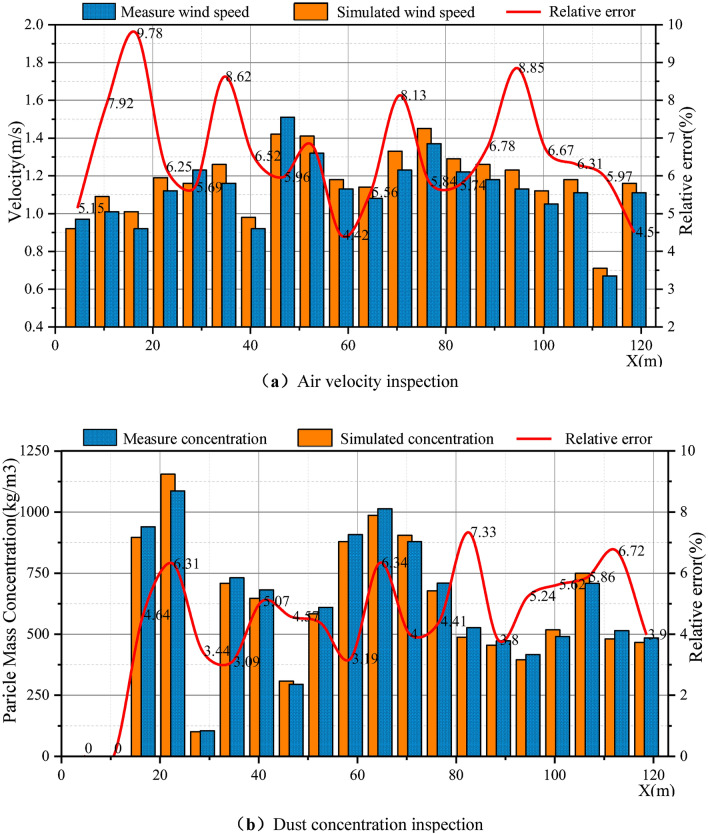
Comparison of numerical simulation and field measurement data of the fully mechanized mining face [(**a**) Verification of air velocity; (**b**) verification of dust concentration).

To verify the feasibility and accuracy of the simulation results, data measurements were performed on a fully mechanized mining face with a face inclination of 40°. The TSI-9545 was used to measure the airflow velocity and AKFC. A -92A dust sampler was used to measure the dust concentration. Because of the actual work situation on site to reduce the occurrence of accidents, the measurement range was concentrated on the sidewalk. The height of the breathing belt was used as an example for data comparison. A total of 20 air velocity and dust concentration observation points were set, and the coordinates were (4.6,1.7,2.7), (10.6,1.7,2.7), (16.6,1.7,2.7), (22.6,1.7,2.7), (28.6,1.7,2.7), (34.6,1.7,2.7), (40.6,1.7,2.7), (46.6,1.7,2.7), (52.6,1.7,2.7), (58.6,1.7,2.7), (64.6,1.7,2.7), (70.6,1.7,2.7), (76.6,1.7,2.7), (82.6,1.7,2.7), (88.6,1.7,2.7), (94.6,1.7,2.7), (100.6,1.7,2.7), (106.6,1.7,2.7), (112.6,1.7,2.7), and (118.6,1.7,2.7), with an interval of 6 m. Each measuring point was measured multiple times, and the average value was compared with the simulation results for verification. The results in Fig. 3 show that the relative error between the numerical simulation results of dust concentration and air velocity and the field-measured data is less than 10%. This finding indicates that the numerical simulation results can objectively and accurately reflect the actual situation of the field, which proves the model established in this study. The model can accurately predict on-site conditions and analyze related motion laws, which have a theoretical guiding role in improving the comprehensive management of working dust.

## Simulation result analysis

### Airflow characteristics of working faces with different inclination angles

**Figure 4:**
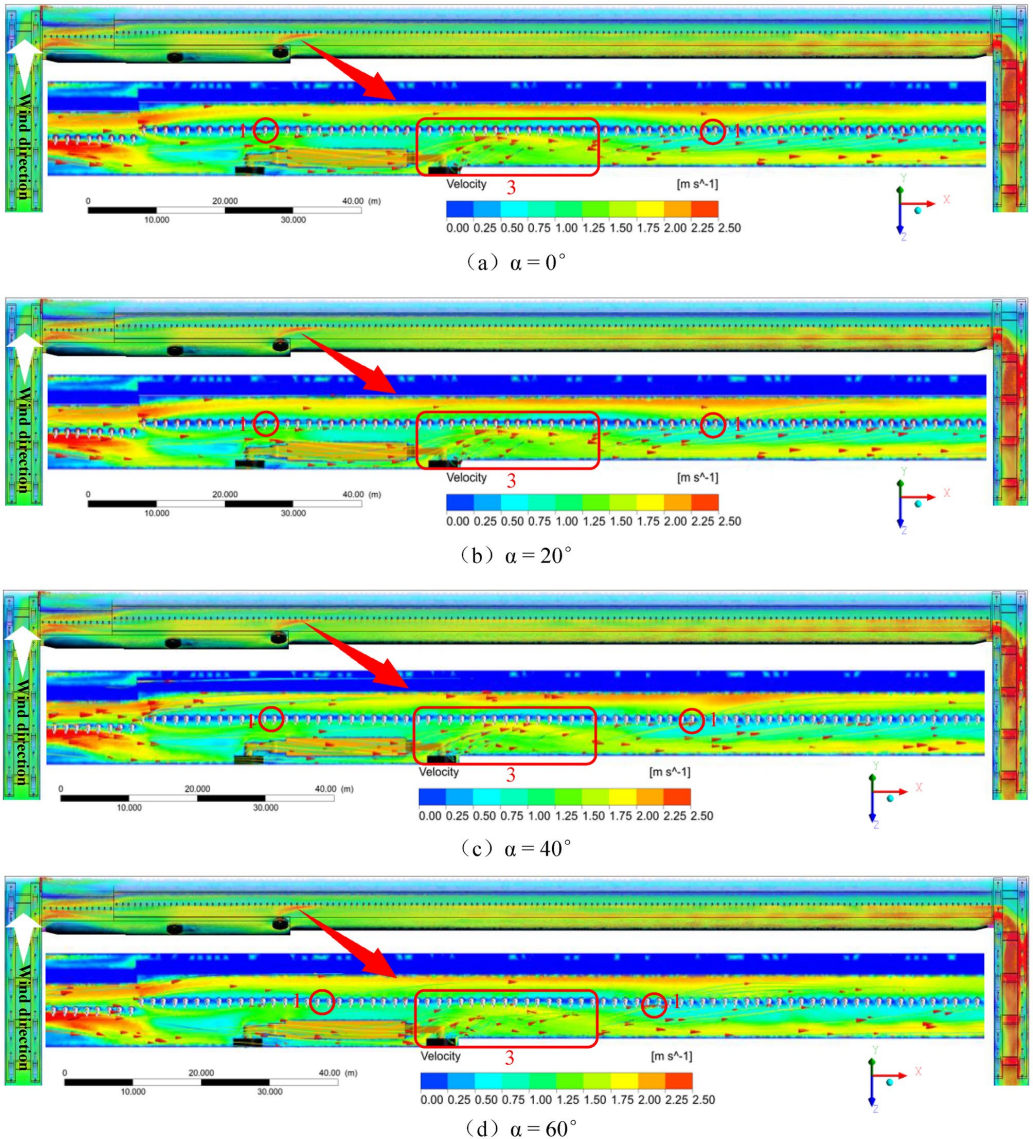
Wind flow renderings at different inclination angles (α = 0°, 20°, 40°, 60°).

The airflow characteristics of the coal mining face under “U”-shaped ventilation conditions are shown in Fig. 4. The color represents the air velocity, the line represents the flow trajectory of the air current, the arrow represents the vector direction of the speed, and the legend is the measurement scale of the air velocity.

1. At 0° working face inclination, the wind flow trajectory is offset at X = 22.5 m, Z = 1.5–2.5 m, and at the sidewalk X = 34.3–64.2 m, Z = 1.1–1.9 m; it forms a width of approximately 0.67 m, the air velocity is approximately 1.81–2.16 m/s due to the disturbance of the turbulent wind cut by the shearer drum; the wind flow flows into the unexploited zone at an angle of 25°–30° and is at the hydraulic support pillar X = 50.2 m. It flows into the sidewalk, which causes the local width of the high-speed wind current belt to reach 1.2 m.
2. At 20° working face inclination, the airflow trajectory is offset at X = 23.1 m, Z = 1.6–2.6 m, and at the sidewalk X = 32.2–61.3 m, Z = 1.1–1.8 m; the width is approximately 0.62 m; the air velocity is approximately 1.82–2.23 m/s due to the disturbance of the turbulent wind cutting by the shearer drum; the wind flow flows into the unexploited zone at an angle of 27°–34° and flow into the sidewalk again at the hydraulic support pillar X = 49.6 m, which causes the local width of the high-speed wind current belt to reach 1.05 m.
3. At 40° working face inclination, the wind flow trajectory is offset at X = 24.4 m, Z = 1.9–2.9 m, and at the sidewalk X = 31.4–59.9 m, Z = 1.1–1.7 m; the width is approximately 0.55 m; the air velocity is approximately 1.82–2.24 m/s due to the disturbance of the turbulent wind cut by the shearer drum; the wind flow flows into the unexploited zone at an angle of 35°–45° and flow into the sidewalk again at the hydraulic support pillar X = 48.3 m, which causes the local width of the high-speed wind current belt to reach 0.64 m.
4. At a 60° working face inclination, the airflow trajectory is offset at X = 27.7 m, Z = 2.05–3 m, and at the sidewalk X = 29.8–58.1 m, Z = 1.1–1.65 m, and the formation width is approximately 0.45. m. The air velocity is approximately 1.83–2.25 m/s. Because of the disturbance of the shearer drum cutting turbulent wind, the wind flow flows into the unexploited zone at an angle of 45°–50° and flows into the sidewalk again at the hydraulic support pillar X = 43.2 m, resulting in the local width of the high-speed wind current belt reaching 0.57.

In summary, the airflow in the air inlet lane is divided into sidewalks and cable duct airflows after entering the working face. The inflection points of the cable duct airflow into the sidewalk under different working face inclination angles differ, and the wind flow deflects twice as the inclination of the working face increases. The distance between the turning points is gradually reduced; simultaneously, with the increase in the inclination of the working face, the wind flow induced by the turbulent wind is cut, the upward inclination angle into the unexploited zone gradually increases, and the width of the high-speed wind flow zone is reduced from 1.2 to 0.57 m, but the maximum air velocity gradually increases from 2.16 to 2.25 m/s.

### Analysis of dust dispersion law

#### Law of dust dispersion and migration on a time scale

**Figure 5:**
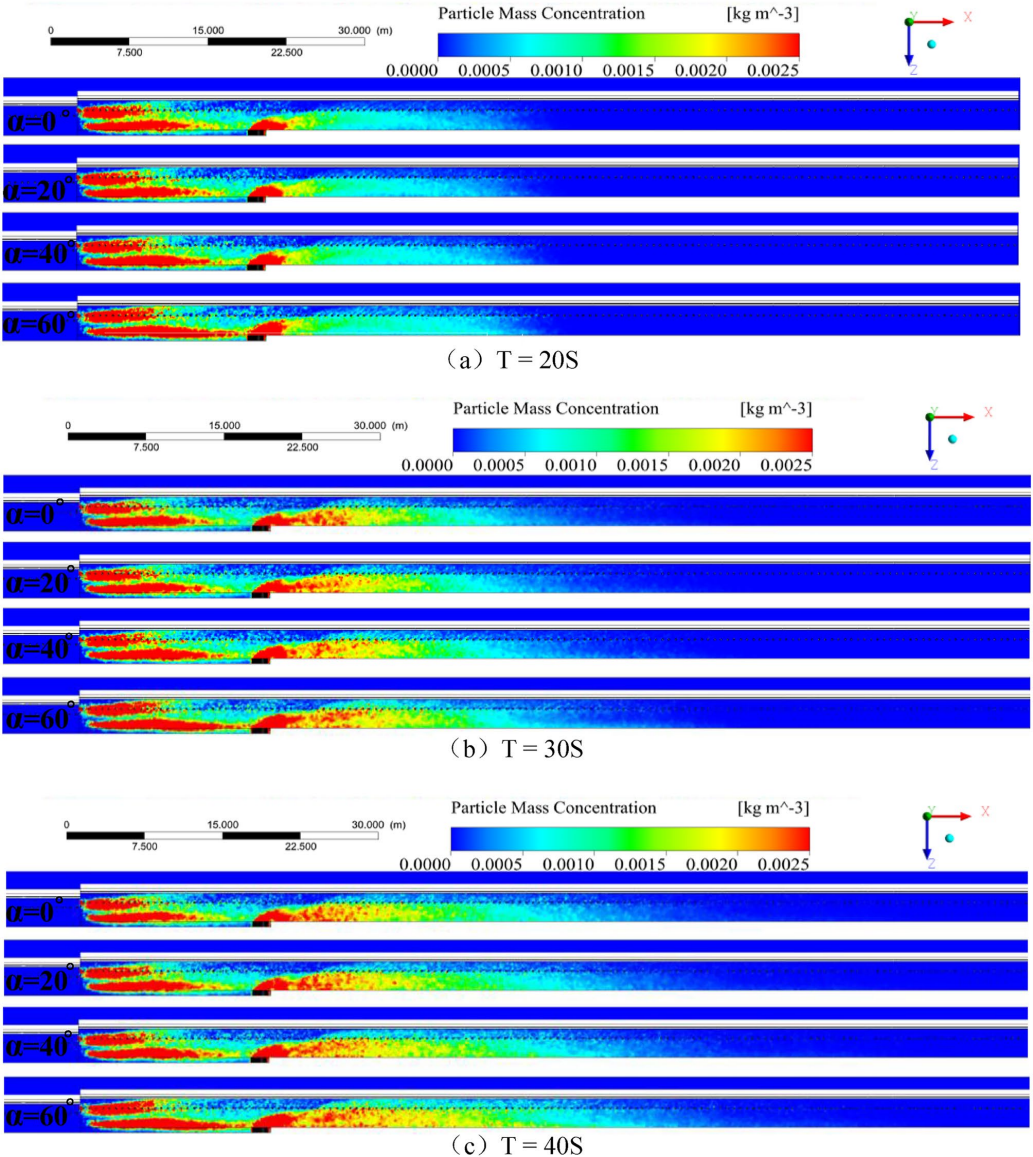

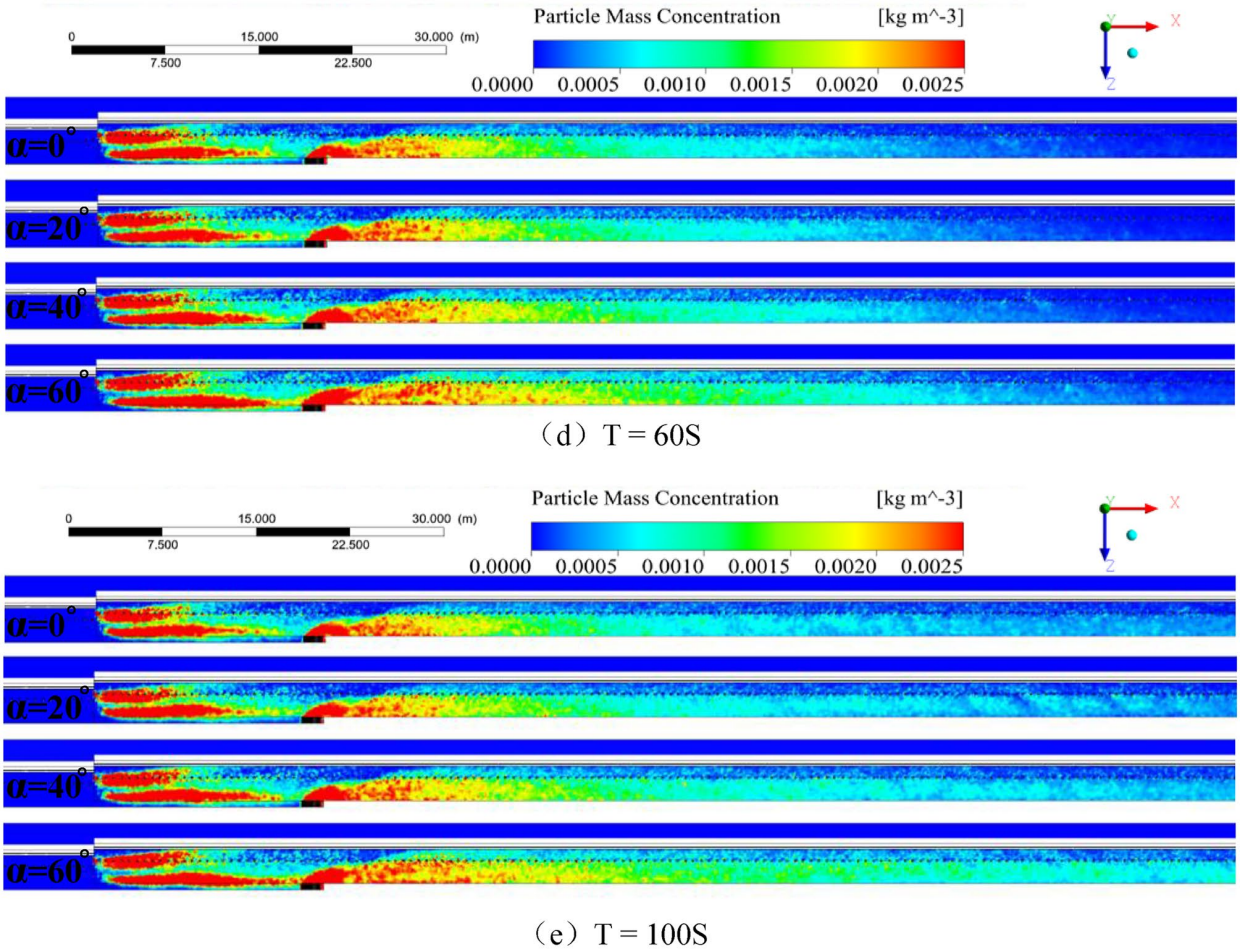
Time chart of dust dispersion distribution at working face Y = 2.8 m (T = 20, 30, 40, 60, 100S).

The plane Y = 2.8 m between the roof and the front drum of the shearer, and the time scale of the fully mechanized mining face, was analyzed. The dust dispersion and migration law is shown in Fig. 5, where the color represents the dust mass concentration, and the legend shows the measurement scale of dust mass concentration. The steps of the analysis were as follows:

After the dust is generated, it gradually diffuses downwind of the working face under the influence of the wind current. Additionally, greater the inclination of the working face, greater the dust dispersion range. The dust source of the moving frame is the same as the airflow, and it is divided into two parts in the working surface: the working space of the cable trough and the sidewalk space. When T = 20–40S, the range of high-concentration dust sources remains basically unchanged under the inclination of each working face. When the time lasts to T = 60–100S, the dust on the sidewalk side is affected by the wind flow and moves in the negative direction of Y. In migration, the dust in the cable trough moves in the Y direction, and the movement trend increases with an increase in the inclination of the working surface. Moreover, the dust produced by drum cutting is disturbed by the cutting turbulent wind and diffuses to the sidewalk. When T = 100S, the dust dispersion state tends to be stable.

The steps of the comparative analysis of the migration law of the cutting dust are as follows:

1. At a working face inclination of 0°, near hydraulic prop X = 41.52 m, dust begins to gradually spread to the sidewalk, which forms dust clusters with a length of approximately 21.93 m, an average width of approximately 1.3 m, and an average concentration of approximately 634.56 mg/m^3^. With the increase in height, clusters begin to show “spot-like” dust clusters at Z = 2.7 m.
2. At a working face inclination of 20°, near hydraulic prop X = 41.26 m, dust begins to gradually spread to the sidewalk, forming dust with a length of approximately 33.38 m, an average width of approximately 1.54 m, and an average concentration of approximately 746.04 mg/m^3^ on the sidewalk. “Spot-like” dust clusters begin to show at Z = 2.74 m.
3. At a working face inclination of 40°, near hydraulic prop X = 41.22 m, initially dust gradually spreads to the sidewalk, forming dust with a length of approximately 42.47 m, an average width of approximately 1.74 m, and an average concentration of approximately 823.47 mg/m^3^. “Spot-like” dust clusters begin to show at Z = 2.8 m.
4. At a working face inclination of 60°, near hydraulic prop X = 40.02 m, initially dust gradually spreads to the sidewalk, forming dust clusters with a length of approximately 65.04 m, an average width of 1.9 m, and an average concentration of approximately 910.56 mg/m^3^ on the sidewalk, which filled the entire sidewalk.

In summary, as the inclination of the working face increases, the dust in the space is closer to the shearer when the dust enters the sidewalk; the length, width, and height of the high-concentration dust cluster formed on the working face will increase accordingly. Additionally, the dust mass concentration increased from 634.56 to 910.56 mg/m^3^. The reason for this finding is that with the increase in the inclination angle of the working face, the upward inclination angle of the wind flow after cutting the turbulent wind and system ventilation increases, and the air velocity increases. which is conducive to the movement of dust, but not for dust to settle; thus, greater the inclination of the working surface, greater the proportion of dust in the entire working space.

#### Law of dust dispersion and migration at the spatial scale

**Figure 6:**
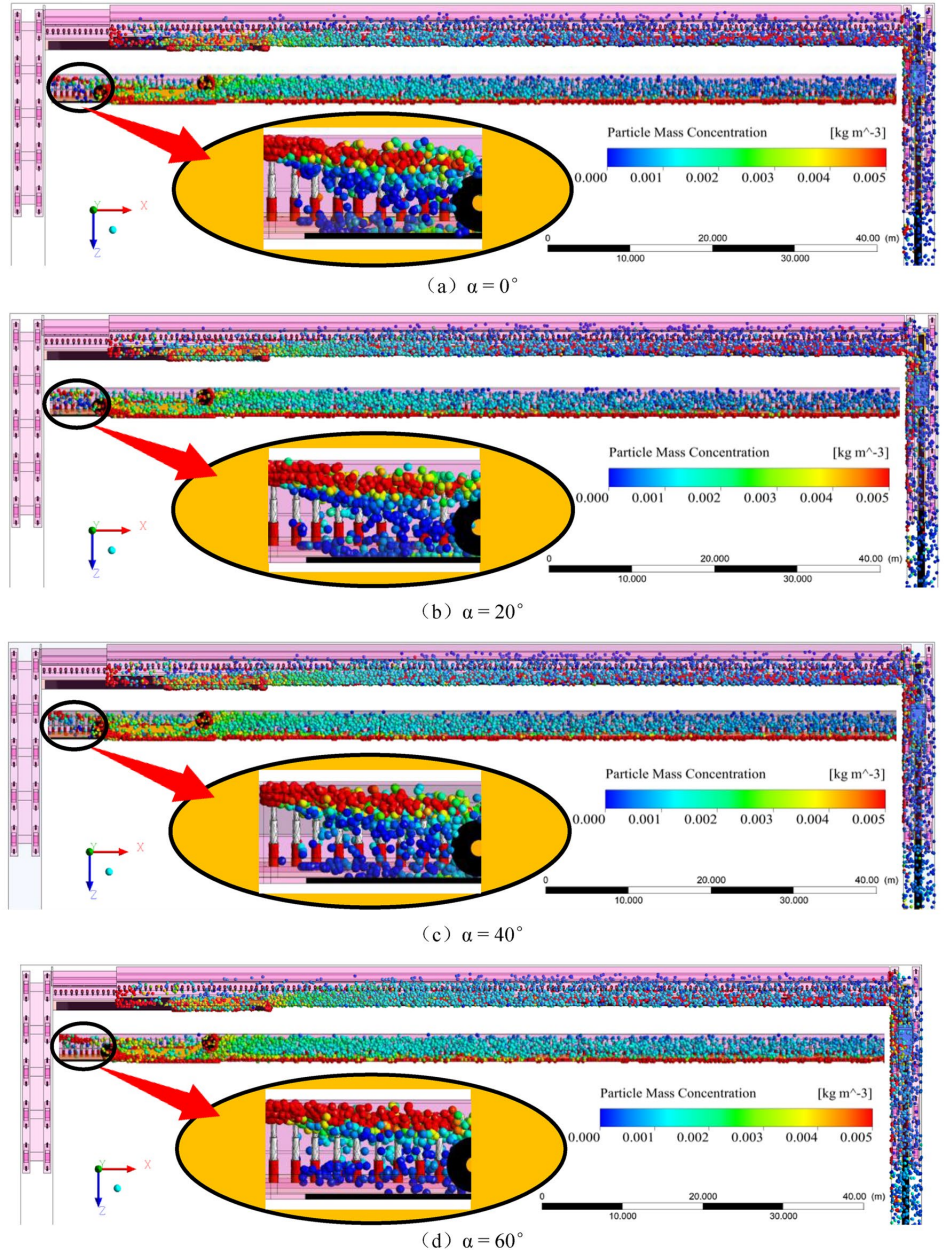
Dust particle dispersion spatial distribution map (α = 0°, 20°, 40°, 60°).

Figure 6 shows the spatial distribution of dust particles in coal mining under the “U”-shaped ventilation condition. dust particle concentration is depicted in different colors and the size of the spheres represents the dust particle diameter. The size of the dust mass concentration is represented by the aforementioned legend.

The steps of the analysis are as follows:

1. The dust generation from the moving frame, combined with that in Figs. 4 and 5, shows that the high-concentration dust group at the moving frame is divided into two parts. The dispersion speed of the high-concentration dust group on the sidewalk was lower than that on the side of the cable trough, because the air velocities on both sides of the hydraulic support pillar differ here, and the air velocity on the side of the cable trough is greater than the air velocity on the sidewalk, which is conducive to the diffusion of dust. With the continuous increase in the inclination of the working face, the tendency for the settlement of high-concentration dust clusters is significantly reduced.
2. When the inclination of the working face is 0°, a high-concentration dust cluster with an average dust mass concentration of 1064.26 mg/m^3^ will form in the zone of X = 34.6–64.56 m, Y = 3.6–5.9 m, Z = 1.8–2.7 m; when the working face inclination is 20°, a high-concentration dust mass with an average dust mass concentration of 1098.44 mg/m^3^ will form in the zone of X = 34.6–70.06 m, Y = 3.3–5.9 m, Z = 1.8–2.75 m; when the working face inclination is 40°, at X = 34.6–83.71 m, Y = 2.7–5.9 m, Z = 1.8–2.75 m, a high-concentration dust mass with an average dust mass concentration of 1108.28 mg/m^3^ will be formed; at a working face inclination of 60°, at X = 34.6–112.47 m, Y = 2.3–5.9 m, Z = 1.8–2.8 m zone forms a high-concentration dust cluster with an average dust mass concentration of 1075.73 mg/m^3^. Hence, when the inclination of the working surface increases, the range of high-concentration dust clusters, suspension time, and settlement range gradually increase.

In summary, this result is due to the speed of dust generated by gravity in space, the speed of wind generation, and the speed of the part of the dust generated by the drum cutting turbulent wind. The three speeds are combined in the velocity vector. However, due to the small dust mass, the vertical velocity generated by gravity can be ignored, When the velocity generated by the cutting turbulent wind remains the same, the greater the inclination angle of the working surface, the greater the angle between the velocity of the dust along the X axis and the working surface, and the greater the sum velocity of the dust and the inclination angle of the working surface. In this case, greater the inclination angle, the dust effect is more evident, the suspension time of the dust on the working surface is higher, and the dispersion distance increases.

#### Analysis of the change in dust concentration in the respiratory zone height

**Figure 7:**
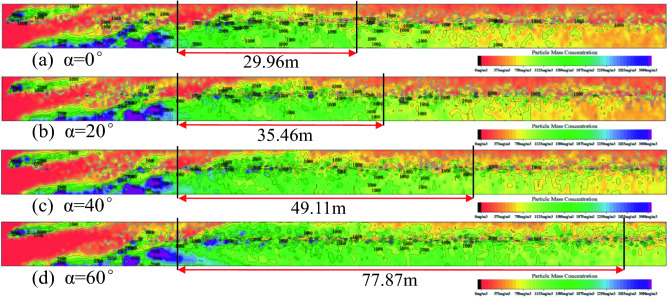
Contour map of dust mass concentration distribution at the height of the respiratory zone (α = 0°, 20°, 40°, 60°).

**Figure 8:**
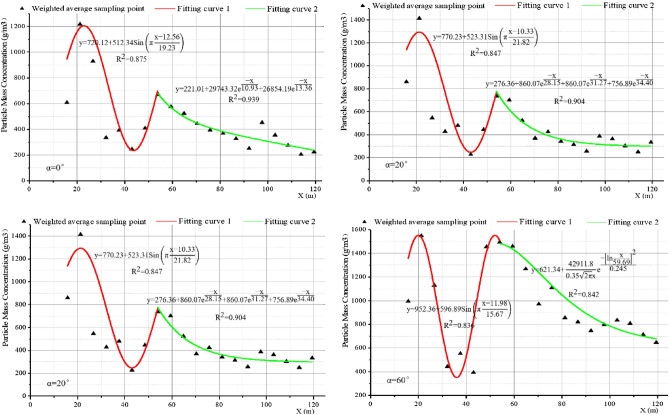
Fitting curve diagram of the sidewalk dust dispersion trend under different working face inclination angles (α = 0°, 20°, 40°, 60°).

The contour map of the dust mass concentration distribution at the height of the working face breathing zone under the condition of “U” ventilation is shown in Fig. 7, where the color represents the dust mass concentration, and the size of the dust mass concentration is represented by the legend at the bottom right. The black closed-loop lines in Fig. 7 are the dust mass concentration contour lines. Figure 8 shows the fitting curve diagram of the dust dispersion trend of the sidewalk at different working face inclination angles, several triangles are scatter plots of dust mass concentration, and the red line and green line represent the first and second segments of the fitting curve.

Greater the inclination of the working surface, greater the dust diffusion zone in the breathing zone. At 0° working face inclination, the high-concentration dust mass is 29.96 m; at 20° working face inclination, the high-concentration dust mass is 35.46 m; at 40° working face inclination, the high-concentration dust mass is 49.11 m; at 60° working face, under the inclination angle, the high-concentration dust mass is 77.87 m; and at different inclination angles of the working face, the tendency of high-concentration dust clusters to diffuse to the sidewalk increases with the increase in the inclination angle of the working face. Therefore, when dust protection is conducted, the influence of the inclination of the working face on the dust dispersion should be considered. Greater the angle of inclination, greater the dust protection range. This makes protection more difficult and increases the threat to the physical and mental health of workers. Based on this background the curve fitting of the sidewalk-respiratory zone dust mass concentration y and the length x of the working face is shown in Fig. 8. The mathematical relationship between the sidewalk-respiratory zone dust mass concentration and the working face length x, for different working face inclination conditions is as follows:

1. When the working face inclination is 0°,
  $$\left\{ {\begin{array}{*{20}l} {y = 720.12 + 512.34{\text{Sin}}\left( {\pi \frac{x - 12.56}{{19.23}}} \right)} \hfill & {13.6 < x < 53.96} \hfill \\ {y = 221.01 + 29743.32e^{{\frac{ - x}{{10.93}}}} + 26854.19e^{{\frac{ - x}{{13.36}}}} } \hfill & {53.96 \le x \le 121.6} \hfill \\ \end{array} } \right.$$
2. When the working face inclination is 20°,
  $$\left\{ {\begin{array}{*{20}l} {y = 770.23 + 523.31{\text{Sin}}\left( {\pi \frac{x - 10.33}{{21.82}}} \right) } \hfill & {13.6 < x < 53.96} \hfill \\ {y = 276.36 + 860.07e^{{\frac{ - x}{{28.15}}}} + 860.07e^{{\frac{ - x}{{31.27}}}} + 756.89e^{{\frac{ - x}{{34.40}}}} } \hfill & {53.96 \le x \le 121.6} \hfill \\ \end{array} } \right.$$
3. When the working face inclination is 40°,
  $$\left\{ {\begin{array}{*{20}l} {y = 804.77 + 542.08{\text{Sin}}\left( {\pi \frac{x - 10.33}{{19.65}}} \right)} \hfill & {13.6 < x < 53.96} \hfill \\ {y = 274.98 + 1805.57e^{{\frac{ - x}{{26.20}}}} + 1815.27e^{{\frac{ - x}{{29.11}}}} + 1789.29e^{{\frac{ - x}{{32.02}}}} } \hfill & {53.96 \le x \le 121.6} \hfill \\ \end{array} } \right.$$
4. When the working face inclination is 60°,
  $$\left\{ {\begin{array}{*{20}l} {{\text{y = 952}}{.36 + 596}{\text{.89Sin}}\left( {{\uppi }\frac{{{\text{x - 11}}{.98}}}{{{15}{\text{.67}}}}} \right)} \hfill & {{13}{\text{.6 < x < 53}}{.96}} \hfill \\ {{\text{y = }}290.84 + 1756.92e^{{\frac{ - x}{{44.47}}}} + 1866.92e^{{\frac{ - x}{{54.36}}}} { }} \hfill & {{53}{\text{.96}} \le {\text{x}} \le {121}{\text{.6}}} \hfill \\ \end{array} } \right.$$

When x = 53.96 is the demarcation point, there is a volatility state before this point, primarily because this point merges with the dust produced by the front drum of the shearer in the middle of the sidewalk, causing the dust concentration at this point to increase suddenly and as the inclination of the working face increases, the dust mass concentration at this point gradually increases, as the inclination of the working surface increases, the dust is more affected by the cutting turbulent wind, and the inclination of the new wind flow formed after mixing with the normal wind flow of the working surface gradually increases, leading to a long period of dust suspension and accumulation. Subsequently, the dust mass concentration shows a downward trend, but as the inclination angle of the working face increases, the downward trend of dust particles gradually decreases, and the suspension time on the working face becomes increases. Through this fitting formula, the focus of dust prevention and control can be appropriately improved, and it has practical significance for the prevention and control of dust and reducing the occurrence of pneumoconiosis.

## Air curtain tracking closed dust control system

**Figure 9:**
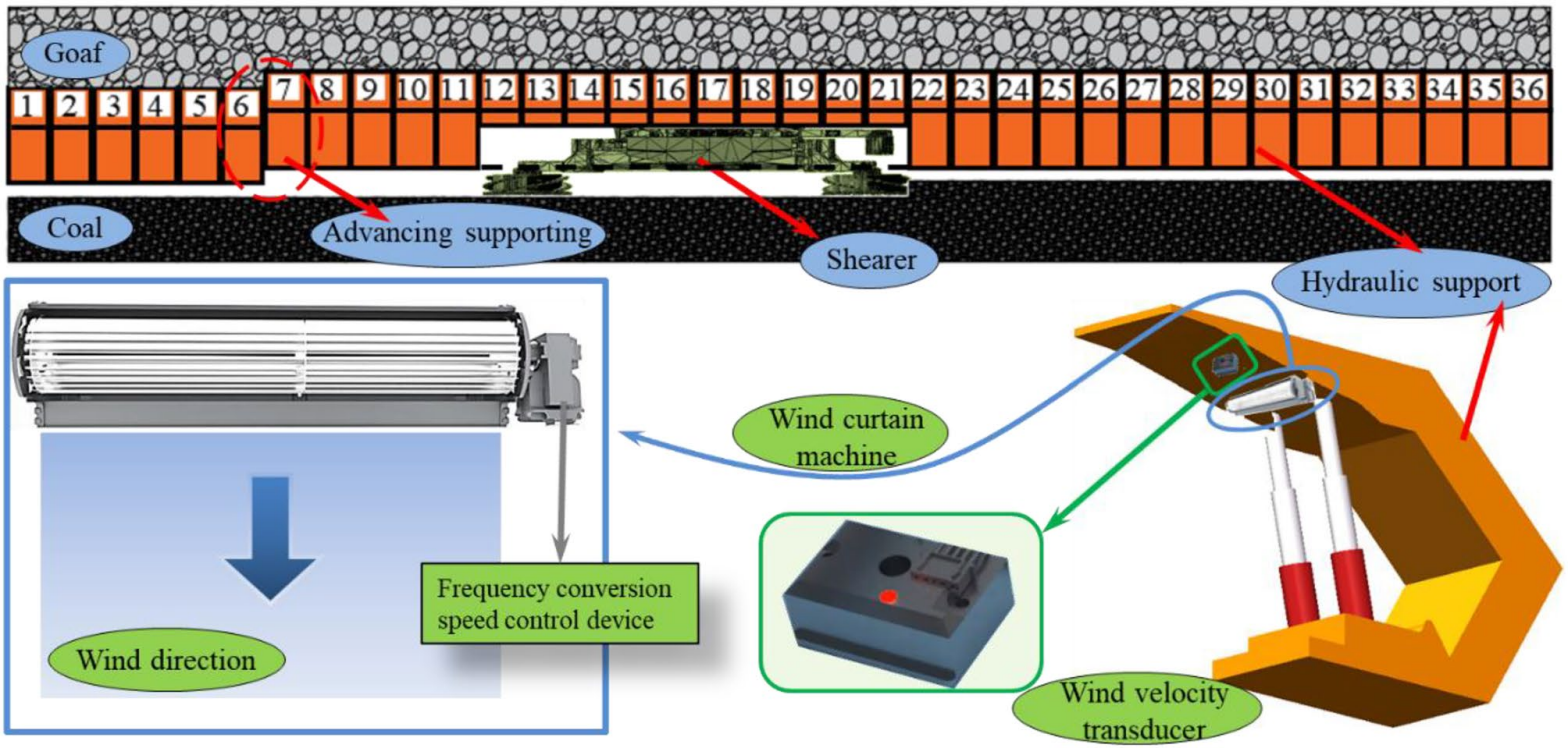
Air curtain tracking closed dust control system equipment diagram.

The numerical simulation results depict that the dust dispersion pattern of the comprehensive mining working face is relatively complicated, due to the influence of the turbulent wind from the shearer's cutting leading to the dust generated by the front drum of the shearer being wrapped in the working face wind flow and entering the unexploited space at different incidence angles, thus causing the high concentration dust clusters in the sidewalk to gather differently. According to this situation, the air curtain tracking closed dust control system is designed as shown in Fig. 9. The wind curtain machine is arranged horizontally along the top of the hydraulic support, and the angle of the wind curtain is regulated by the wind fence to form a barrier wind curtain, so that the sidewalk is isolated from the coal mining operation zone, and the air velocity is detected by the air velocity sensor, and the wind volume of the wind curtain machine is controlled by the frequency conversion speed control device, to achieve better isolation of dust.

**Figure 10:**
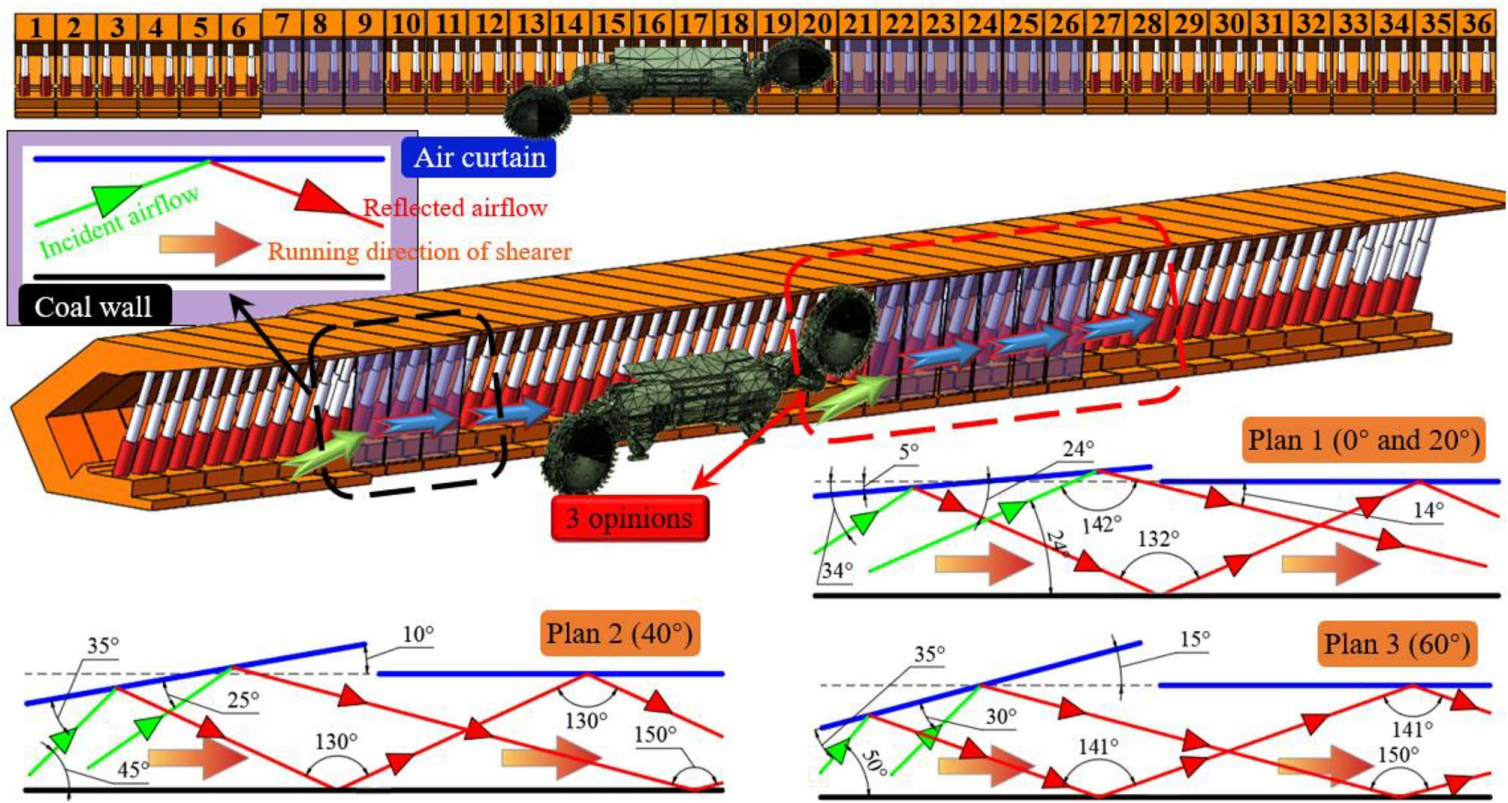
Diagram of wind curtain adjustment.

In the above analysis, the angle of incidence is 25°–30° and 27°–34° for 0° and 20° face inclination respectively, and the air velocity is maintained at 1.81–2.23 m/s. The angle of incidence and the position of dust entering the sidewalk are similar, hence a dust control scheme is set up for both cases, while for 40° and 60° face inclination, the angle of incidence and the position of dust entering the sidewalk differ significantly. Therefore, for different inclination angles of the dust into the sidewalk angle and position, the design of three wind curtain machine dust control program, where two adjacent wind curtain machines with the operation. The wind curtain adjustment diagram is shown in Fig. 10

1. When the wind flow enters the comprehensive mining working face from the inlet lane, the wind flow is diverted, part of which flows into the sidewalk and the other part flows into the cable trough. When the working face inclination is 0° and 20°, the air velocity is small and the wind flow is influenced by the hydraulic pillar, which leads to a smaller angle of wind deflection, and the tendency of the dust carried by the moving frame to enter the sidewalk is small. The dust produced by the front roller of the coal mining machine is at the height of the breathing zone, which is a serious hazard, and is influenced by the cut-off turbulent wind flowing to the sidewalk and unexploited zone at an incidence angle of 25° to 34°, so the wind curtains No. 21, 22, 23, 24, 25 and 26 are turned on, and a wind curtain of + 5° is formed in the running direction of the coal mining machine to adjust the direction of the wind flow, and at the same time, the air velocity sensor is combined to adjust the wind curtain speed is adjusted in combination with the air velocity sensor to change the trend of dust transport to the sidewalk and improve the working environment at the working face.
2. When the working face inclination angle is 40°, the tendency of the dust carried by the wind flow into the sidewalk increases obviously, and the dust carried by the dust is concentrated in the vicinity of the roof, and the diffusion range increases due to the effect of the updraft. The wind curtain is formed in the direction of + 10° to regulate the direction of the wind flow, and the speed of the wind curtain is adjusted in combination with the air velocity sensor to control the trend of dust transport and improve the working environment at the working face.
3. When the angle of inclination of the working face is 60°, the dust is concentrated near the top plate and the tendency of lateral and vertical movement increases greatly, thus the wind curtain No. 7, 8, 10, 11, 13, 14, 16 and 17 are turned on to form a vertical wind curtain to prohibit the wind flow from entering the sidewalk with the dust; the wind flow is subject to the turbulent wind cut by the drum in front of the shearer at an incidence angle of 45° to 50° and flows to the sidewalk and the unexploited zone. When a wide range of highly congested dust clouds are formed, air curtains 21, 22, 23, 24, 25, 26, 27, 28, 29, and 30 are switched on and a curtain of + 15° in the direction of the shearer's operation is formed to regulate the direction of the wind flow, while the speed of the curtain is adjusted in conjunction with the air velocity sensor to control the dust transport trend and improve the working environment at the working face.

## Conclusion

1. With the increase of the working face inclination, the upward inclination of the airflow into the undeveloped zone gradually increases from 25° to 50°, and the maximum wind speed of the sidewalk gradually increases from 2.16 to 2.25 m/s, and the width decreases from 1.2 to 0.57 m.
2. When the inclination of the working face increases, under the influence of cutting turbulent wind and system ventilation, the dust is accelerated to the sidewalk side. Meanwhile, the range of high-concentration dust clusters, suspension time, lateral migration intensity, and deposition area increase to varying degrees; the volume of the dust clusters increases from 62.02 to 202.46 m^3^.
3. Under the working face inclinations of 0°, 20°, 40°, and 60°, When X < 53.96 m, the dust concentration in the sidewalk-breathing zone shows a sine function with the length of the working face, and when X ≥ 53.96 m, it satisfies the exponential decay function.
4. The air curtain tracking closed dust control technology is proposed. According to the different angles of the offset of the wind flow and the gathering position of the high concentration dust mass under the inclination of the working surface, the air curtain angle and air velocity are automatically tracked and controlled by the infrared control device, to control the tendency of the dust transfer to the working face and ensure that the dust is controlled on the cable trough side as much as possible.

## Data Availability

All data generated or analysed during this study are included in this published article.
